# Peritoneal metastasis as a predictive factor for nab-paclitaxel in patients with pretreated advanced gastric cancer: an exploratory analysis of the phase III ABSOLUTE trial

**DOI:** 10.1007/s10120-018-0838-6

**Published:** 2018-05-31

**Authors:** Atsuo Takashima, Kohei Shitara, Kazumasa Fujitani, Keisuke Koeda, Hiroki Hara, Norisuke Nakayama, Shuichi Hironaka, Kazuhiro Nishikawa, Yutaka Kimura, Kenji Amagai, Hirofumi Fujii, Kei Muro, Taito Esaki, Yasuhiro Choda, Toshimi Takano, Keisho Chin, Atsushi Sato, Masahiro Goto, Norimasa Fukushima, Takuo Hara, Nozomu Machida, Manabu Ohta, Narikazu Boku, Masashi Shimura, Satoshi Morita, Wasaburo Koizumi

**Affiliations:** 1 Gastrointestinal Medical Oncology Division, National Cancer Center Hospital, 5-1-1, Tsukiji, Chuo-ku, Tokyo, 104-0045 Japan; 2 Department of Gastrointestinal Oncology, National Cancer Center Hospital East, Kashiwa, Japan; 3 Department of Surgery, Osaka General Medical Center, Osaka, Japan; 4 Department of Surgery, Iwate Medical University School of Medicine, Morioka, Japan; 5 Department of Gastroenterology, Saitama Cancer Center, Ina-machi, Japan; 6 Department of Gastroenterology, Kanagawa Cancer Center, Yokohama, Japan; 7 Clinical Trial Promotion Department, Chiba Cancer Center, Chiba, Japan; 8 Department of Surgery, National Hospital Organization Osaka National Hospital, Osaka, Japan; 9 Department of Surgery, Sakai City Medical Center, Sakai, Japan; 10 Department of Gastroenterology, Ibaraki Prefectural Central Hospital, Kasama, Japan; 11 Department of Clinical Oncology, Jichi Medical University Hospital, Shimotsuke, Japan; 12 Department of Clinical Oncology, Aichi Cancer Center Hospital, Nagoya, Japan; 13 Department of Gastrointestinal and Medical Oncology, National Kyushu Cancer Center, Fukuoka, Japan; 14 Department of Surgery, Hiroshima City Hiroshima Citizens Hospital, Hiroshima, Japan; 15 Department of Medical Oncology, Toranomon Hospital, Tokyo, Japan; 16 Department of Gastroenterology, Cancer Institute Hospital of the Japanese Foundation for Cancer Research, Tokyo, Japan; 17 Department of Medical Oncology, Hirosaki University Graduate School of Medicine, Hirosaki, Japan; 18 Cancer Chemotherapy Center, Osaka Medical College Hospital, Takatsuki, Japan; 19 Department of Surgery, Yamagata Prefectural Central Hospital, Yamagata, Japan; 20 Department of Surgery, Kouseiren Takaoka Hospital, Takaoka, Japan; 21 Division of Gastrointestinal Oncology, Shizuoka Cancer Center, Sunto-gun, Japan; 22 Oncology Center, Hamamatsu University School of Medicine, Hamamatsu, Japan; 23 Data Science Department, Taiho Pharmaceutical. Co., Ltd., Tokyo, Japan; 24 Department of Biomedical Statistics and Bioinformatics, Kyoto University Graduate School of Medicine, Kyoto, Japan; 25 Department of Gastroenterology, Kitasato University School of Medicine, Sagamihara, Japan

**Keywords:** Peritoneal metastasis, Nab-paclitaxel, Solvent-based paclitaxel, Second-line chemotherapy, Predictive factor

## Abstract

**Background:**

In the ABSOLUTE trial, weekly nanoparticle albumin-bound paclitaxel (w-nab-PTX) showed non-inferiority to weekly solvent-based paclitaxel (w-sb-PTX) for overall survival (OS). Thus, w-nab-PTX might be an option for second-line chemotherapy in advanced gastric cancer (AGC). However, predictive factors for efficacies of these agents have not been evaluated.

**Methods:**

Patients previously enrolled in the ABSOLUTE trial were divided into apparent peritoneal metastasis group (PM group) and no apparent peritoneal metastasis group (no PM group) based on baseline imaging evaluated by RECIST ver. 1.1 criteria and amount of ascites. OS, progression-free survival, and overall response rate were compared between two arms in each group.

**Results:**

This study included 240 and 243 patients in the w-nab-PTX and w-sb-PTX arms, respectively. In the PM group, the w-nab-PTX arm (*n* = 88) had longer OS than the w-sb-PTX arm (*n* = 103), and median survival time (MST) of 9.9 and 8.7 months [hazard ratio (HR) 0.63; 95% CI 0.45–0.88; *P* = 0.0060], respectively. In the no PM group, the w-nab-PTX arm (*n* = 140) had shorter OS than the w-sb-PTX arm (*n* = 152), and MST of 11.6 and 15.7 months (HR 1.40; 95% CI 1.06–1.86; *P* = 0.0180), respectively. After adjusting for prognostic factors, the HR for OS in the w-nab-PTX arm versus the w-sb-PTX arm was 0.59 (95% CI 0.42–0.83; *P* = 0.0023; PM group) and 1.34 (95% CI 1.01–1.78; *P* = 0.0414; no PM group), with significant interaction between treatment efficacy and presence of peritoneal metastasis (*P* = 0.0003).

**Conclusions:**

The presence of apparent peritoneal metastasis might be a predictive factor for selecting w-nab-PTX for pretreated AGC patients.

**Trial registration number:**

JapicCTI-132059.

**Electronic supplementary material:**

The online version of this article (10.1007/s10120-018-0838-6) contains supplementary material, which is available to authorized users.

## Introduction

Solvent-based paclitaxel (sb-PTX) is one of the standard second-line chemotherapies for advanced gastric cancer (AGC). Recently, the ABSOLUTE trial compared the efficacy and safety of nanoparticle albumin-bound paclitaxel (nab-PTX) (260 mg/m^2^) of every 3 weeks schedule and weekly nab-PTX (100 mg/m^2^) (w-nab-PTX) with that of weekly sb-PTX (80 mg/m^2^) (w-sb-PTX) in patients with AGC refractory to a fluoropyrimidine-containing chemotherapy regimen [1]. The non-inferiority of w-nab-PTX to w-sb-PTX in overall survival (OS) was confirmed, while that of nab-PTX (every 3 weeks) to w-sb-PTX was not. Based on the ABSOLUTE trial results, w-nab-PTX might be an option for second-line chemotherapy for AGC. However, a predictive factor for selecting either w-sb-PTX or w-nab-PTX is not yet available.

The peritoneum is among the most frequent metastatic sites of AGC. Moreover, the frequency of peritoneal metastasis increases with the clinical course; it is more common in pretreated patients with AGC than in chemotherapy-naïve patients. Several phase II trials showed the promising efficacy of w-sb-PTX for patients with AGC with peritoneal metastasis [2, 3]. A subgroup analysis of the ABSOLUTE trial suggested that w-nab-PTX showed more favorable efficacy than w-sb-PTX in patients with peritoneal metastasis. Furthermore, the effect of w-nab-PTX was correlated with the amount of ascites [1]. W-nab-PTX is speculated to have advantages over w-sb-PTX depending on the severity of peritoneal metastasis.

However, criteria for evaluating the severity of peritoneal metastasis have not been established; neither the presence of peritoneal lesions detected by imaging nor the amount of ascites appropriately reflect the severity of peritoneal metastasis. Some patients were diagnosed with peritoneal metastasis via staging laparoscopy before the initiation of first-line chemotherapy and without peritoneal lesions or ascites detectable by imaging before the start of second-line chemotherapy. While some patients have ascites without visible peritoneal lesions such as peritoneal nodules, thickening of the mesenterium, and bowel deformity or obstruction, visible peritoneal metastasis is sometimes not associated with ascites. Therefore, neither the presence of visible peritoneal lesions nor ascites alone is adequate for selecting which regimen, w-nab-PTX or w-sb-PTX, is optimal for the patient. Moreover, the subgroup analysis of the ABSOLUTE trial was not adjusted by prognostic factors between the two arms.

In this study, we compared the efficacy of w-nab-PTX and w-sb-PTX according to the severity of peritoneal metastasis based on visible peritoneal lesions and amount of ascites.

## Methods

### Patients and methods

A total of 742 patients were enrolled in the ABSOLUTE trial between March 2013 and May 2015. We selected patients allocated to the w-nab-PTX and w-sb-PTX arms, because w-nab-PTX showed non-inferiority to w-sb-PTX in terms of OS in the ABSOLUTE trial. The presence of peritoneal metastasis was a stratification factor. Patients in the w-nab-PTX arm received 100 mg/m^2^ intravenous nab-PTX over 30 min on days 1, 8, and 15 every 4 weeks (1 cycle), while those in the w-sb-PTX arm received 80 mg/m^2^ intravenous sb-PTX over 60 min after pre-medication with steroids and histamine H_2_ receptor antagonists on days 1, 8, and 15 every 4 weeks (1 cycle).

The review board at each participating institution approved this trial, which was conducted according to the Declaration of Helsinki, International Conference on Harmonisation, and Good Clinical Practice. All patients provided written informed consent.

### Classification of peritoneal metastasis

We used two factors to classify peritoneal metastasis based on baseline imaging. First was the presence of detectable peritoneal lesions by investigator-judged imaging, either measurable or non-measurable according to RECIST ver. 1.1, such as peritoneal nodules, thickening of the mesenterium, and bowel deformity or obstruction. Second was the amount of ascites evaluated by investigator-judged imaging. Small and large amounts of ascites were defined as “ascites limited to the pelvic cavity” and “ascites from the pelvis extending continuously to the upper abdomen”, respectively. A moderate amount of ascites was termed “ascites other than small or large ascites”. Patients were categorized as having “massive ascites” (presence of large or moderate ascites) or “no massive ascites” (presence of small ascites or no ascites).

Using these two categories, the subjects were classified into four groups: group A comprised patients with no detectable peritoneal lesions and no massive ascites; group B is comprised of patients with detectable peritoneal lesions and no massive ascites; group C is comprised of patients with no detectable peritoneal lesions and massive ascites; and group D is comprised patients of with detectable peritoneal lesions and massive ascites. Furthermore, patients in groups B, C, and D who had apparent peritoneal metastasis were unified to the apparent peritoneal metastasis group (PM group), while patients in group A who did not have apparent peritoneal metastasis were assigned to the no apparent peritoneal metastasis group (no PM group).

### Statistical analysis

The efficacy endpoints of this study were OS, progression-free survival (PFS), and overall response rate (ORR). Survival curves were estimated using the Kaplan–Meier method. The hazard ratio (HR) and *P* value for OS and PFS of the w-nab-PTX group compared with the w-sb-PTX group were calculated using the Cox proportional hazard model. ORR was analyzed in patients with at least one measurable lesion at baseline. We used Fisher’s exact test to compare the ORR between the two treatment groups and the logistic regression model to assess the interaction for ORR among the groups. To adjust the confounding factors and assess interaction between treatment groups (w-nab-PTX and w-sb-PTX) and subgroups of peritoneal metastasis (peritoneal metastasis and non-peritoneal metastasis), we used the other prognostic factors as covariates such as age, performance status, histological type, previous gastrectomy, type of treatment failure with previous chemotherapy, and duration of prior chemotherapy for multivariate analysis. The confidence coefficient for the confidence interval of the median time and HR for OS and PFS, and ORR was set to 95% (*P* < 0.05). All analyses were performed using SAS version 9.2.

## Results

A total of 483 patients were analyzed in this study. Among them, 240 and 243 belonged to the w-nab-PTX and w-sb-PTX groups, respectively. All 483 patients were included in the full analysis set for the primary analysis in the ABSOLUTE trial. The patient characteristics are shown in Table 1; the classification into groups A–D is shown in Supplementary Fig. 1.

**Table 1:** Baseline characteristics of patients in the four groups

	Group A (*N* = 292)		Group B (*N* = 118)		Group C (*N* = 38)		Group D (*N* = 35)		Total
	w-nab-PTX (*N* = 152)	w-sb-PTX (*N* = 140)	w-nab-PTX (*N* = 54)	w-sb-PTX (*N* = 64)	w-nab-PTX (*N* = 19)	w-sb-PTX (*N* = 19)	w-nab-PTX (*N* = 15)	w-sb-PTX (*N* = 20)	
Age (years), *n* (%)									
< 65	58 (38)	61 (44)	22 (41)	30 (47)	7 (37)	13 (68)	8 (53)	11 (55)	210
≥ 65	94 (62)	79 (56)	32 (59)	34 (53)	12 (63)	6 (31)	7 (47)	9 (45)	273
Sex, *n* (%)									
Female	38 (25)	31 (22)	11 (20)	19 (30)	8 (42)	12 (63)	5 (33)	5 (25)	129
Male	114 (75)	109 (78)	43 (80)	45 (70)	11 (58)	7 (37)	10 (67)	15 (75)	354
ECOG performance status, *n* (%)									
0	115 (76)	112 (80)	29 (54)	32 (50)	12 (63)	11 (58)	12 (80)	13 (65)	336
1	35 (23)	25 (18)	25 (46)	32 (50)	7 (37)	7 (37)	3 (20)	7 (35)	141
2	2 (1)	3 (2)	0	0	0	1 (5)	0	0	6
Histological type, *n* (%)									
Diffuse	73 (48)	66 (47)	37 (69)	36 (56)	15 (79)	16 (84)	12 (80)	14 (70)	269
Intestinal	79 (52)	74 (53)	17 (31)	28 (44)	4 (21)	3 (16)	3 (20)	5 (25)	213
Unknown	0	0	0	0	0	0	0	1 (5)	1
Previous gastrectomy, *n* (%)									
No	73 (48)	64 (46)	21 (39)	28 (44)	5 (26)	8 (42)	10 (67)	11 (55)	220
Yes	79 (52)	76 (54)	33 (61)	36 (56)	14 (74)	11 (58)	5 (33)	9 (45)	263
Number of organs with metastases, *n* (%)									
< 2	87 (57)	76 (54)	14 (26)	12 (19)	9 (47)	15 (79)	4 (27)	5 (25)	222
≥ 2	65 (43)	64 (46)	40 (74)	52 (81)	10 (53)	4 (21)	11 (73)	15 (75)	261
Previous use of docetaxel, *n* (%)									
No	139 (91)	127 (91)	48 (89)	57 (89)	16 (84)	19 (100)	13 (87)	16 (80)	435
Yes	13 (9)	13 (9)	6 (11)	7 (11)	3 (16)	0	2 (13)	4 (20)	48
Previous chemotherapy regimens, *n* (%)									
Fluoropyrimidine monotherapy	58 (38)	54 (39)	23 (43)	25 (39)	11 (58)	5 (26)	5 (33)	2 (10)	183
Doublet chemotherapy	83 (55)	76 (54)	28 (52)	34 (53)	6 (32)	14 (74)	9 (60)	15 (75)	265
Triplet chemotherapy	11 (7)	10 (7)	3 (6)	5 (8)	2 (11)	0	1 (7)	3 (15)	35
Duration of previous chemotherapy (months), *n* (%)									
< 6	77 (51)	60 (43)	22 (41)	18 (28)	6 (32)	7 (37)	7 (47)	5 (25)	202
≥ 6	75 (49)	80 (57)	32 (59)	46 (72)	13 (68)	12 (63)	8 (53)	15 (75)	281
Recurrence during adjuvant chemotherapy	43 (28)	45 (32)	14 (26)	15 (23)	7 (37)	4 (21)	2 (13)	2 (10)	132
Progressive disease during first-line chemotherapy, *n* (%)	109 (72)	95 (68)	40 (74)	49 (77)	12 (63)	15 (79)	13 (87)	18 (90)	351

Group A: patients with no detectable peritoneal lesions and no massive ascites, group B: patients with detectable peritoneal lesions and no massive ascites, group C: patients with no detectable peritoneal lesions and massive ascites, group D: patients with detectable peritoneal lesions and massive ascites

*w-nab-PTX* weekly nanoparticle-bound paclitaxel, *w-sb-PTX* weekly solvent-based paclitaxel

**Fig. 1:**
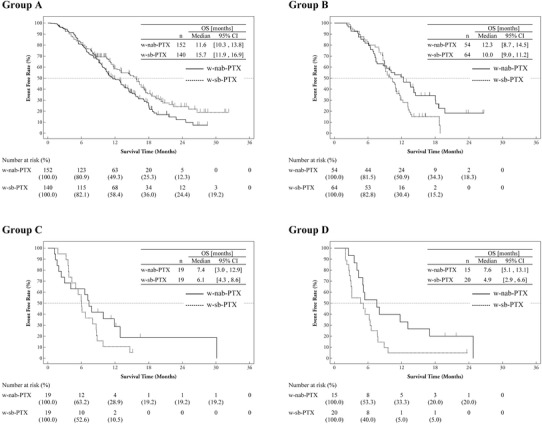
Kaplan–Meier plot of overall survival by subgroup (4-group version). Group A: patients with no detectable peritoneal lesions and no massive ascites, group B: patients with detectable peritoneal lesions and no massive ascites, group C: patients with no detectable peritoneal lesions and massive ascites, group D: patients with detectable peritoneal lesions and massive ascites. *w-nab-PTX* weekly nanoparticle-bound paclitaxel, *w-sb-PTX* weekly solvent-based paclitaxel, *OS* overall survival

The OS and PFS are summarized in Table 2 and the survival curves in Fig. 1. The HRs for the OS of the w-nab-PTX arm compared with the w-sb-PTX arm in groups A, B, C, and D were 1.40 (95% CI 1.06–1.86; *P* = 0.018), 0.64 (95% CI 0.42–1.00; *P* = 0.046), 0.66 (95% CI 0.32–1.34; *P* = 0.245), and 0.47 (95% CI 0.22–1.00; *P* = 0.044), respectively. The HRs for the PFS of the w-nab-PTX arm compared with the w-sb-PTX arm in groups A, B, C, and D were 1.02 (95% CI 0.80–1.30; *P* = 0.888), 0.62 (95% CI 0.42–0.93; *P* = 0.020), 0.77 (95% CI 0.38–1.54; *P* = 0.448), and 0.43 (95% CI 0.20–0.89; *P* = 0.019), respectively. The results for the ORR are summarized in Supplementary Table 1. The ORRs were not significantly different between the w-nab-PTX and w-sb-PTX arms except in group B [45.5% (15/33) vs. 15.9% (7/44), *P* = 0.0057].

**Table 2:** Summary of overall survival and progression-free survival

Group^a^	*n*	OS			PFS		
		Median OS (months) (95% CI)	HR (95% CI)	*P* value^b^	Median PFS (months) (95% CI)	HR (95% CI)	*P* value^b^
A							
w-nab-PTX	152	11.6 (10.3–13.8)	1.40 (1.06–1.86)	0.018	4.9 (3.8–5.6)	1.02 (0.80–1.30)	0.888
w-sb-PTX	140	15.7 (11.9–16.9)			3.8 (3.7–4.7)		
B							
w-nab-PTX	54	12.3 (8.7–14.5)	0.64 (0.42–1.00)	0.046	6.0 (5.1–7.6)	0.62 (0.42–0.93)	0.020
w-sb-PTX	64	10.0 (9.0–11.2)			3.7 (3.5–4.1)		
C							
w-nab-PTX	19	7.4 (3.0–12.9)	0.66 (0.32–1.34)	0.245	4.5 (1.9–7.1)	0.77 (0.38–1.54)	0.448
w-sb-PTX	19	6.1 (4.3–8.6)			4.3 (2.7–5.6)		
D							
w-nab-PTX	15	7.6 (5.1–13.1)	0.47 (0.22–1.00)	0.044	4.0 (3.4–8.1)	0.43 (0.20–0.89)	0.019
w-sb-PTX	20	4.9 (2.9–6.6)			2.6 (1.7–3.8)		

*w-nab-PTX* weekly nanoparticle-bound paclitaxel, *w-sb-PTX* weekly solvent-based paclitaxel, *OS* overall survival, *PFS* progression-free survival, *HR* hazard ratio

^a^Group A: patients with no detectable peritoneal lesions and no massive ascites, group B: patients with detectable peritoneal lesions and no massive ascites, group C: patients with no detectable peritoneal lesions and massive ascites, group D: patients with detectable peritoneal lesions and massive ascites

^b^Log rank test

In the PM group, the w-nab-PTX arm showed a higher ORR than the w-sb-PTX arm: 40.0% (16/40) for w-nab-PTX and 16.4% (9/55) for w-sb-PTX (*P* = 0.0172). The ORR in the no PM group was similar in the two treatment arms: 30.0% (33/110) for w-nab-PTX and 28.1% (32/114) for w-sb-PTX (*P* = 0.7702). In a multivariate analysis adjusting for prognostic factors, the odds ratio for ORR was 3.945 (95% CI 1.402–11.102; *P* = 0.0093) in the PM group and 1.086 (95% CI 0.593–1.991; *P* = 0.7890) in the no PM group. The interaction for ORR after adjusting for prognostic factors was significant (*P* = 0.0174).

**Fig. 2:**
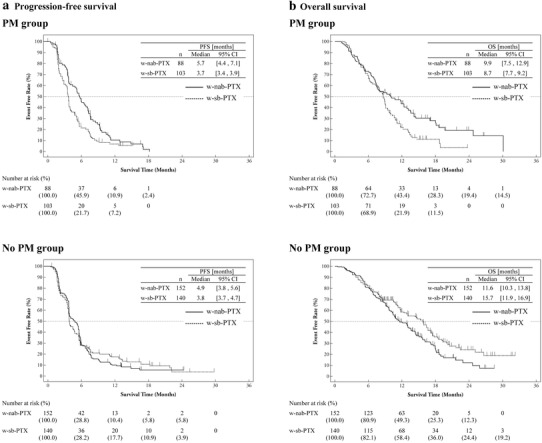
Kaplan–Meier plot of overall survival and progression-free survival for the reclassified subgroup (2-group version). **a** Progression-free survival. **b** Overall survival. *w-nab-PTX* weekly nanoparticle-bound paclitaxel, *w-sb-PTX* weekly solvent-based paclitaxel, *PM group* the apparent peritoneal metastasis group, *no PM group* the no apparent peritoneal metastasis group, *PFS* progression-free survival, *OS* overall survival

The median PFS in the PM group was 5.7 months (95% CI 4.4–7.1) and 3.7 months (95% CI 3.4–3.9) for the w-nab-PTX arm (HR 0.62; 95% CI 0.46–0.85; *P* = 0.0024). In the no PM group (group A), the PFS was similar between the two treatment arms (median PFS: 4.9 months for w-nab-PTX and 3.8 months for w-sb-PTX; HR 1.02; 95% CI 0.80–1.30; *P* = 0.888) (Fig. 2a). In a multivariate analysis adjusting for prognostic factors, the HR for PFS of the w-nab-PTX arm compared with the w-sb-PTX arm was 0.54 (95% CI 0.39–0.75; *P* = 0.0002) in the PM group and 0.93 (95% CI 0.72–1.20; *P* = 0.5734) in the no PM group. The interaction for PFS after adjusting for prognostic factors was significant between the PM and no PM groups (*P* = 0.0191).

In the PM group, 52/88 (59.1%) patients treated with w-nab-PTX and 66/103 (64.1%) patients treated with w-sb-PTX received post-study treatment (*P* = 0.551). In the no PM group, 101/152 patients (66.4%) treated with w-nab-PTX and 111/140 patients (79.3%) treated with w-sb-PTX received post-study treatment (*P* = 0.018) (Supplementary Table 2).

The median OS in the PM group was 9.9 months (95% CI 7.5–12.9) and 8.7 months (95% CI 7.7–9.2) for the w-nab-PTX arm and the w-sb-PTX arm (HR 0.63; 95% CI 0.45–0.88; *P* = 0.0060), respectively (Fig. 2b). In a multivariate analysis adjusting for prognostic factors, the HR for OS of the w-nab-PTX arm compared with the w-sb-PTX arm was 0.59 (95% CI 0.42–0.83; *P* = 0.0023) in the PM group and 1.34 (95% CI 1.01–1.78; *P* = 0.0414) in the no PM group. The interaction for OS was significantly different between the PM and no PM groups after adjusting for prognostic factors (*P* = 0.0003). For sensitivity analysis, we added the multivariate analysis of OS and PFS to include the imbalanced factors of sex and the number of metastatic organs in addition to the original covariates. The results of the additional analysis were similar to those of analyses undertaken without adjusting for sex and the number of metastatic organs (data not shown).

## Discussion

To the best of our knowledge, this study is the first to assess the efficacy of w-nab-PTX and w-sb-PTX based on peritoneal metastasis. We showed that w-nab-PTX yielded better OS, PFS, and ORR than w-sb-PTX in patients with apparent peritoneal metastasis.

In this study, we divided the patients into four groups using two categories, the presence of detectable peritoneal lesions by imaging and the amount of ascites. Among the four groups, group D, containing patients with both detectable peritoneal lesions and massive ascites, showed the poorest prognosis, while group A, containing patients without detectable peritoneal lesions or massive ascites, showed the best prognosis. Moreover, the prognosis of patients in groups B and C were between groups A and D. These results suggested that our classification of peritoneal metastasis based on the two categories was an accurate reflection of peritoneal metastasis severity.

Furthermore, the HRs for both OS and PFS in the w-nab-PTX arm compared with the w-sb-PTX arm were the lowest in group D, indicating a better efficacy of w-nab-PTX in that group. However, the HR was the highest in group A, indicating poor efficacy of w-nab-PTX. Furthermore, the efficacy of w-nab-PTX in groups B and C were in the middle of groups A and D, indicating good efficacy of w-nab-PTX in such groups. A comparison of ORR between the w-nab-PTX and the w-sb-PTX arms showed a similar relationship among all four groups. These results suggest that the efficacy of w-nab-PTX compared with w-sb-PTX may relate to peritoneal metastasis severity in patients with AGC.

In the treatment of patients with AGC, the presence or absence of apparent peritoneal metastasis is important for selecting anti-tumor agents, particularly as second- or later-line chemotherapy. Because of its toxicities, irinotecan is contraindicated for patients with AGC who have severe peritoneal metastasis. We unified groups B, C, and D as the PM group. The interaction *P* values for OS, PFS, and ORR between the PM and no PM groups were consistently less than 0.05 even after adjusting for background prognostic factors. The presence or absence of apparent peritoneal metastasis might be a predictive marker for selecting either w-nab-PTX or w-sb-PTX as second-line chemotherapy for patients with AGC.

Differences in drug formulations between nab-PTX and sb-PTX might explain the significantly better efficacy of w-nab-PTX in patients with AGC; sb-PTX contains polyethoxylated castor oil as a solvent and alcohol, whereas nab-PTX is a solvent-free albumin-bound form of PTX. Albumin has a high affinity for hydrophobic drugs including PTX [4] and is transported across the endothelial barrier of blood vessels through binding to the gp60 albumin receptor and activation of caveolae-mediated endothelial transcytosis [5–7]. Simulations based on population pharmacokinetic modeling showed that the tissue distribution of nab-PTX was more dependent upon an active transport mechanism, i.e., endothelial transcytosis for drug distribution into tissues than that of sb-PTX [8]. A preclinical study in rabbits, in which nab-PTX and sb-PTX were intraperitoneally administered, showed that nab-PTX penetrated the peritoneum tissue better than sb-PTX [9]. Moreover, a previous preclinical study that compared intravenous administration of nab-PTX with intraperitoneal administration of sb-PTX in a peritoneal metastasis mouse model reported that intravenous nab-PTX demonstrated an equivalent effect of reducing ascites and peritoneal tumors to intraperitoneal sb-PTX at equal doses [10]. Furthermore, the reduction rate of ascites in w-nab-PTX was better than in w-sb-PTX (24.8 vs. 13.5%) in the ABSOLUTE trial [11] and a greater efficacy for peritoneal metastasis would lead to longer survival.

In the no PM group, although the w-nab-PTX arm showed comparable PFS and ORR with the w-sb-PTX arm, the w-sb-PTX arm showed longer OS than the w-nab-PTX arm. The median OS of w-sb-PTX (15.7 months) in this study is remarkably longer than that in other clinical trials of second-line chemotherapy for patients with AGC, and is better than studies of first-line chemotherapy for AGC, which have reported OSs ranging from 6 to 13.8 months [12–17]. While this favorable OS reported with w-sb-PTX may have some bias such as hidden imbalance of other prognostic factors, the fact that more patients treated with w-sb-PTX received post-study treatment might be one of the reasons for the difference in OS between the two arms.

Our study had some limitations. The subgroups analysis in this study was not pre-planned; thus, the results should be interpreted with caution. The mechanism causing the difference in efficacy for peritoneal metastasis between w-nab-PTX and w-sb-PTX cannot be completely explained biologically, although the additional analysis between every 3 weeks nab-PTX and w-sb-PTX in the ABSOLUTE trial using our reclassified subgroup also suggested the same trend in terms of HR for OS and PFS (Supplementary Table 3). The standard second-line chemotherapy for AGC is the combination of ramucirumab with sb-PTX. While, phase II studies of ramucirumab with nab-PTX (JapicCTI-153088, NCT02317991) showed promising activity and manageable toxicities [18]. However, it is still unclear whether the results in the present study for selecting w-nab-PTX or w-sb-PTX are applicable to the combination with ramucirumab. Future prospective studies of nab-PTX with ramucirumab for patients with AGC with peritoneal metastasis/ascites are needed. The West Japan Oncology Group is planning a randomized trial comparing w-nab-PTX with ramucirumab and w-sb-PTX with ramucirumab for AGC with peritoneal metastasis.

In conclusion, our study suggests that the presence or absence of apparent peritoneal metastasis might be a predictive factor for selecting w-nab-PTX or w-sb-PTX in patients with pretreated AGC.

## Electronic supplementary material

Below is the link to the electronic supplementary material.

Supplementary material 1 (DOCX 84 KB)
