# Supplementary material for: Peritoneal metastasis as a predictive factor for nab-paclitaxel in patients with pretreated advanced gastric cancer: an exploratory analysis of the phase III ABSOLUTE trial
Source: Gastric Cancer. 2018 May 31;22(1):155–63. doi: 10.1007/s10120-018-0838-6 (PMC6315007; doi:10.1007/s10120-018-0838-6)
Supplement: Supplementary file 1 — Supplementary material 1 (DOCX 84 KB) [file 10120_2018_838_MOESM1_ESM.docx]

# **Supplementary Materials**

**Journal name:**

Gastric Cancer

**Article title:**

Peritoneal metastasis as a predictive factor for nab-paclitaxel in patients with pretreated advanced gastric cancer: An exploratory analysis of the phase III ABSOLUTE trial

**Author names:**

Atsuo Takashima, Kohei Shitara, Kazumasa Fujitani, Keisuke Koeda, Hiroki Hara, Norisuke Nakayama, Shuichi Hironaka, Kazuhiro Nishikawa, Yutaka Kimura, Kenji Amagai, Hirofumi Fujii, Kei Muro, Taito Esaki, Yasuhiro Choda, Toshimi Takano, Keisho Chin, Atsushi Sato, Masahiro Goto, Norimasa Fukushima, Takuo Hara, Nozomu Machida, Manabu Ohta, Narikazu Boku, Masashi Shimura, Satoshi Morita, Wasaburo Koizumi

**Corresponding author:**

Dr. Atsuo Takashima

Gastrointestinal Medical Oncology Division, National Cancer Center Hospital, Tokyo, Japan

E-mail: [atakashi@ncc.go.jp](mailto:atakashi@ncc.go.jp)

## **Supplementary Figure 1. Groups classified**


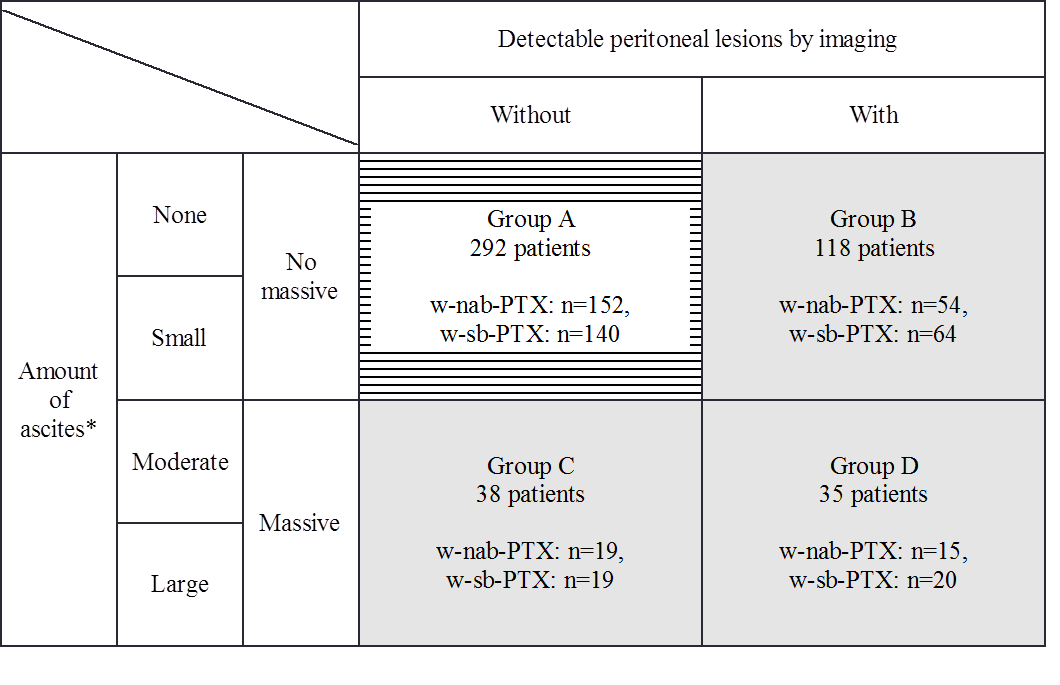


*None indicates no ascites on imaging, small indicates that ascites were limited to pelvic cavity on imaging, large indicates that ascites extend continuously from the pelvis to the upper abdomen, and moderate indicates another variation of ascites than specified here.

: no apparent peritoneal metastases group (no PM group): group A

: apparent peritoneal metastases group (PM group): groups B+C+D:

Abbreviations: weekly solvent-based paclitaxel; w-nab-PTX, weekly solvent-based paclitaxel; w-sb-PTX

## **Supplementary Table 1. Summary of overall response rate**

|  | w-nab-PTX | w-sb-PTX | *P* Value* |
| --- | --- | --- | --- |
| Group A. Patients with no detectable peritoneal lesions and no massive ascites | | | |
| (CR+PR)/Total (%) | 33/110 (30.0) | 32/114 (28.1) |  |
| 95% CI | [21.6, 39.5] | [20.1, 37.3] | 0.7702 |
| Group B. Patients with detectable peritoneal lesions and no massive ascites | | | |
| (CR+PR)/Total (%) | 15/33 (45.5) | 7/44 (15.9) |  |
| 95% CI | [28.1, 63.7] | [6.6, 30.1] | 0.0057 |
| Group C. Patients with no detectable peritoneal lesions and massive ascites | | | |
| (CR+PR)/Total (%) | 0/3 (0) | 0/2 (0) |  |
| 95% CI | [-, -] | [-, -] | 1.0000 |
| Group D. Patients with detectable peritoneal lesions and massive ascites | | | |
| (CR+PR)/Total (%) | 1/4 (25.0) | 2/9 (22.2) |  |
| 95% CI | [0.6, 80.6] | [2.8, 60.0] | 1.0000 |

*Fisher’s exact test

w-nab-PTX, weekly nanoparticle-bound paclitaxel; w-sb-PTX, weekly solvent-based paclitaxel.

## **Supplementary Table 2. Summary of post-study treatment**

|  |  | Apparent peritoneal metastasis group | | |  | No apparent peritoneal metastasis group | | |
| --- | --- | --- | --- | --- | --- | --- | --- | --- |
|  |  | w-nab-PTX (*n*=88) | w-sb-PTX (*n*=103) | *P* Value* |  | w-nab-PTX (*n*=152) | w-sb-PTX (*n*=140) | *P* Value* |
|  |  | *n* (%) | *n* (%) |  |  | *n* (%) | *n* (%) |  |
| Post-study treatment |  |  |  |  |  |  |  |  |
| No |  | 36 (40.9) | 37 (35.9) | F:0.551 |  | 51 (33.6) | 29 (20.7) | F:0.018 |
| Yes |  | 52 (59.1) | 66 (64.1) |  |  | 101 (66.4) | 111 (79.3) |  |
| Chemo/immuno/hormonal treatment |  |  |  |  |  |  |  |  |
| No |  | 36 (40.9) | 37 (35.9) | F:0.551 |  | 52 (34.2) | 29 (20.7) | F:0.013 |
| Yes |  | 52 (59.1) | 66 (64.1) |  |  | 100 (65.8) | 111 (79.3) |  |
| Surgery |  |  |  |  |  |  |  |  |
| No |  | 85 (96.6) | 101 (98.1) | F:0.663 |  | 149 (98.0) | 132 (94.3) | F:0.126 |
| Yes |  | 3 (3.4) | 2 (1.9) |  |  | 3 (2.0) | 8 (5.7) |  |
| Other treatment |  |  |  |  |  |  |  |  |
| No |  | 87 (98.9) | 103 (100.0) | F:0.461 |  | 151 (99.3) | 140 (100.0) | F:1.000 |
| Yes |  | 1 (1.1) | 0 (0.0) |  |  | 1 (0.7) | 0 (0.0) |  |
| Number of chemo/immuno/hormonal treatment lines |  |  |  |  |  |  |  |  |
| None |  | 36 (40.9) | 37 (35.9) | W:0.477 |  | 52 (34.2) | 29 (20.7) | W:0.019 |
| 3rd line |  | 36 (40.9) | 45 (43.7) |  |  | 61 (40.1) | 63 (45.0) |  |
| 4th line |  | 11 (12.5) | 14 (13.6) |  |  | 23 (15.1) | 32 (22.9) |  |
| 5th line |  | 5 (5.7) | 5 (4.9) |  |  | 13 (8.6) | 13 (9.3) |  |
| 6th line |  | 0 (0.0) | 1 (1.0) |  |  | 0 (0.0) | 2 (1.4) |  |
| 7th line |  | 0 (0.0) | 1 (1.0) |  |  | 2 (1.3) | 0 (0.0) |  |
| 8th line |  | 0 (0.0) | 0 (0.0) |  |  | 1 (0.7) | 1 (0.7) |  |

*F: Fisher’s exact test, W: Wilcoxson rank sum test.

w-nab-PTX, weekly nanoparticle-bound paclitaxel; w-sb-paclitaxel, weekly solvent-based paclitaxel.

## **Supplementary Table 3. Summary of overall survival and progression free survival for the reclassified subgroup (2-group version; w-sb-PTX versus every 3 weeks nab-PTX).**

| Group |  | *n* |  | OS | | |  | PFS | | |
| --- | --- | --- | --- | --- | --- | --- | --- | --- | --- | --- |
|  |  |  |  | Median OS (months) [95% CI] | HR [95% CI] | *P* Value^*^ |  | Median PFS (months) [95% CI] | HR [95% CI] | *P* Value^*^ |
| PM group | every 3 weeks nab-PTX  w-sb-PTX | 79  103 |  | 7.9 [6.5–9.9]  8.7 [7.7–9.2] | 0.91 [0.66–1.26] | 0.567 |  | 3.4 [2.1–3.8]  3.7 [3.4–3.9] | 1.04 [0.76–1.42] | 0.815 |
| no PM group | every 3 weeks nab-PTX  w-sb-PTX | 164  140 |  | 11.4 [10.3–14.0]  15.7 [11.9–16.9] | 1.29 [0.98–1.70] | 0.068 |  | 4.1 [3.7–5.4]  3.8 [3.7–4.7] | 1.07 [0.84–1.37] | 0.561 |

*Log rank test.

PM group, apparent peritoneal metastasis group; no PM group, no apparent peritoneal metastasis group; nab-PTX, nanoparticle-bound paclitaxel; w-sb-PTX, weekly solvent-based paclitaxel; OS, overall survival; PFS, progression-free survival; HR, hazard ratio.
